# Current and cumulative night shift work and subclinical atherosclerosis: results of the Gutenberg Health Study

**DOI:** 10.1007/s00420-016-1150-6

**Published:** 2016-07-05

**Authors:** S. Jankowiak, E. Backé, F. Liebers, A. Schulz, J. Hegewald, S. Garthus-Niegel, M. Nübling, S. Blankenberg, N. Pfeiffer, K. J. Lackner, M. Beutel, M. Blettner, T. Münzel, P. S. Wild, A. Seidler, S. Letzel, U. Latza

**Affiliations:** 1 Division Work and Health, Federal Institute for Occupational Safety and Health (BAuA), Noeldnerstr. 40-42, 10317 Berlin, Germany; 2 Preventive Cardiology and Preventive Medicine, Department of Medicine 2, University Medical Center Mainz, Johannes Gutenberg University Mainz, Langenbeckstr. 1, 55131 Mainz, Germany; 3 Faculty of Medicine, Institute and Policlinic of Occupational and Social Medicine, TU Dresden, Fetscherstr. 74, 01307 Dresden, Germany; 4 Department of Psychosomatics and Health Behaviour, Norwegian Institute of Public Health, 0403 Oslo, Norway; 5 FFAS, Freiburg Research Centre for Occupational and Social Medicine, Bertoldstr. 27, 79098 Freiburg, Germany; 6 Department of General and Interventional Cardiology, University Heart Center Hamburg, Martinistr. 52, 20251 Hamburg, Germany; 7 Department of Ophthalmology, University Medical Center Mainz, Johannes Gutenberg-Universität Mainz, Langenbeckstr. 1, 55131 Mainz, Germany; 8 Institute for Clinical Chemistry and Laboratory Medicine, University Medical Center, Johannes Gutenberg-Universität Mainz, Langenbeckstr. 1, 55131 Mainz, Germany; 9 Department of Psychosomatic Medicine and Psychotherapy, University Medical Center Mainz, Johannes Gutenberg-Universität Mainz, Langenbeckstr. 1, 55131 Mainz, Germany; 10 Institute of Medical Biostatistics, Epidemiology and Informatics, University Medical Centre, Johannes Gutenberg-Universität Mainz, Obere Zahlbacher Straße 69, 55131 Mainz, Germany; 11 DZHK (German Center for Cardiovascular Research), Partner Site Rhein-Main, Langenbeckstr. 1, 55131 Mainz, Germany; 12 Center of Thrombosis and Hemostasis (CTH), University Medical Center Mainz, Langenbeckstr. 1, 55131 Mainz, Germany; 13 Institute of Occupational, Social and Environmental Medicine, University Medical Center of the Johannes Gutenberg University Mainz, Obere Zahlbacher Straße 67, 55131 Mainz, Germany

**Keywords:** Night shift work, Arterial stiffness, Vascular function, Intima media thickness, Occupation, Population based

## Abstract

**Purpose:**

The study examines the association between exposure to current and cumulative night shift work and subclinical parameters of atherosclerosis.

**Methods:**

Participants of a population-based cohort study (the Gutenberg Health Study, *N* = 15,010) aged 35–64 years were examined at baseline (2007–2012). Investigations included measurements of arterial stiffness, vascular function [reactive hyperaemia (RH) index], and intima media thickness (IMT). Also, a complete job history (including up to 15 periods), occupational exposures, a variety of lifestyle, and dispositional variables were enquired.

**Results:**

Night shift work was performed by 1071 out of 8065 currently employed individuals. The strongest association after adjustment for age, sex, job complexity level, being a manager, overtime work, and noise appeared for more than 660 night shifts within the last 10 years and a significantly increased arterial stiffness of 0.33 m/s. This reflects a 4 % flow velocity increase for individuals with more than 660 night shifts compared to non-night workers. Regarding the entire professional life, night shift workers showed a significantly decreased vascular function by −0.054 RH index points by using the same adjustment. IMT values did not differ statistically from non-night workers. Lifestyle and dispositional factors showed an influence on all used subclinical atherosclerosis parameters.

**Conclusions:**

The cross-sectional results demonstrate an association between night work and detrimental changes in the atherosclerotic process. The association is more pronounced with more years in night shift and is partly explained by lifestyle and dispositional factors. Longitudinal analyses are necessary to confirm the results.

## Introduction

 Shift work is associated with circadian disruption, disturbs sleep and social life, and modifies disease risk factors (Puttonen et al. 2010; Ramin et al. 2015). Thus, shift work could potentially contribute to various chronic diseases including cardiovascular diseases (CVDs) (Frost et al. 2009; Wang et al. 2011). A considerable number of workers are concerned since the prevalence of shift work is estimated to range roughly between 15 and 20 % across the EU and in the USA (Eurofound 2012; IARC 2010; Statistisches Bundesamt 2014).

Several studies have investigated the association between shift work and CVD. A meta-analysis (Vyas et al. 2012) confirms a modest association between shift work and myocardial infarction, describing a risk ratio of 1.23 [95 % confidence interval (CI) 1.15–1.31]. Night shift workers have been found to face the highest risk (risk ratio 1.41, 95 % CI 1.13–1.76). Shift work was not, however, associated with increased rates of mortality. The association of shift work and hypertension is still under debate (Esquirol et al. 2011). Some studies prove an association with the onset and progression of hypertension (e.g. de Bacquer et al. 2009; Kubo et al. 2013; Oishi et al. 2005).

Research to detect associations between shift work and cardiovascular health is challenging because of the long follow-up time that is needed to cover late outcomes such as myocardial morbidity (e.g. angina pectoris or myocardial infarction) or mortality. There may be a subsequent difficulty concerning a healthy worker effect since workers who cannot tolerate night work may change to day work.

One approach of tackling these issues is to include surrogate parameters that describe early subclinical changes in vascular structure and function, such as arterial stiffness, peripheral vascular function, or intima media thickness (IMT). The parameters differ both biologically regarding their potential for reversibility of the changes (e.g. after treatment), the interference with concurrent endogenous or exogenous factors (e.g. diurnal cycle), and methodologically regarding size and location of the blood vessel examined, as well as the type of measurement technique applied. Thus, it is important to measure both structural and functional outcomes because they may illuminate different mechanisms on the complex pathways to CVD [see (Puttonen et al. 2009)].

All of these surrogate parameters are discussed in recent publications as risk prediction markers of coronary events (Lorenz et al. 2012; Vlachopoulos et al. 2010; Yeboah et al. 2009). Yet, it remains uncertain to which extent the measurement of these markers adds to traditional instruments of risk prediction (de Ruijter et al. 2009), and whether the measures are independent or complementary (Lemos et al. 2015).

Measurements of selected subclinical parameters have been included in cross-sectional analyses on shift work (Chen et al. 2010; Kantermann et al. 2013; Suessenbacher et al. 2011) and one recent cohort study (Wang et al. 2015). So far, the stiffness index has only been investigated in one pilot study that considered exposure to shift work (Kantermann et al. 2013).

Against this background, we examined the association between night shift work and early cardiovascular markers in a population-based sample of employees from the baseline examination of the prospective Gutenberg Health Study (GHS). The objectives of these cross-sectional analyses were to investigate associations and dose–response relationships between (1) current and (2) cumulative exposure to night shift work and adverse effects on both structural (carotid IMT) and functional (arterial stiffness and peripheral vascular function) non-invasive parameters of subclinical atherosclerosis.

## Methods

### Study population

GHS is an ongoing single-centre population-based cohort study designed and conducted at the University Medical Centre in Mainz, Germany. Its main emphasis is placed on cardiovascular functioning and the improvement of vascular risk stratification (Wild et al. 2012). The sampling was based on 35- to 74-year-old residents of the City of Mainz and the district of Mainz-Bingen, randomly drawn and equally stratified by sex and residence for each decade of age. The baseline examinations were performed between 2007 and 2012 on 15,010 enroled persons. The 5-h-long examination included a variety of interviews, blood sampling, and clinical examinations. The recruitment efficacy proportion and the cooperation proportion in the baseline were 55.5 and 70.0 %, respectively (unpublished data).

For the purposes of these analyses, individuals were excluded who (1) were older than 64 years (retirement age) at the day of the interview (*n* = 3753), (2) did not provide any occupational information (*n* = 57), or (3) stated to have never been employed in their lifetime (for at least 1 year in part-time); (*n* = 617). Out of the eligible 10,583 individuals between 35 and 64 years of age, complete information about confounders and covariates was available for 10,475 subjects included in the analyses. Subjects were either currently employed (*n* = 8065) or not working at present (*n* = 2410; 23 %; e.g. unemployed, on parental leave, or in training).

Individuals with a self-reported diagnosis of CVD (i.e. coronary heart disease, myocardial infarction, stroke, or peripheral artery occlusive disease) were excluded in sensitivity analyses. To determine the influence of a history of cancer on the effects, sensitivity analyses were also conducted by additionally excluding individuals who reported a cancer diagnosis in the past.

### Definition of exposure

Subjects were requested to specify every past occupational period including their occupational titles, as well as their current job. A maximum of 15 occupational periods could be covered within an interview. On average participants reported four periods. Within each of the occupational periods, the number of night shifts per month was enquired. Night shift work was defined as working hours between 11 p.m. and 5 a.m.

Based on this information, two main variables, current and cumulative night shift, were formed. The variable ‘current night shift’ was subdivided into a binary variable (night shift yes or no), and a numerical variable reflecting the number of current night shifts per month.

Cumulative night shifts were calculated in different steps. Within one occupational period, the reported number of night shifts per month was multiplied by eleven (since approximately 1 month of annual leave is standard in Germany) times the number of years in that period. Subsequently, the single periods spent with job tasks at night were summed up. The following final categorisation was used: ‘no exposure’ = 0 nights, ‘low exposure’ = 1–220 nights, ‘medium exposure’ = 221–660 nights, and ‘high exposure’ = more than 660 nights. The cut-off value of 220 equals the accumulation of one work year in night shift work (i.e. 250 annual working days − 30 days of annual leave). The described categories of cumulative number of night shifts were considered for (a) the last 10 years, and (b) the entire working life.

### Outcomes

The measurements used for investigations related to occupational exposures included arterial stiffness, vascular function, and IMT, bio-molecular examinations of fibrinogen and C-reactive protein, and clinical examinations (e.g. blood pressure, hypertension). For the present paper, the following early parameters of CVD were selected as outcomes in agreement with the GHS study protocol.

Arterial stiffness was measured as stiffness index examined by digital photoplethysmography (Pulse Trace PCA 2™, Micro Medical Ltd., currently CareFusion). The change in the volume pulse is registered via the pulse-dependent absorption of infrared light (The Reference Values for Arterial Stiffness’ Collaboration 2010). The stiffness index is measured in m/s (body height/peak-to-peak time) and considered to be continuous. A high-flow velocity expresses inelastic (stiff) arterial blood vessels.

Vascular function of the resistance vessels is estimated by reactive hyperaemia index (RHI) that works as a continuous variable. The pulse amplitude was recorded by volume plethysmography with the non-invasive Endo-PAT2000™ device (Itamar Medical, Caesarea, Israel) that detects plethysmographically pressure changes in the finger tips caused by the arterial pulse. After occlusion of the brachial artery, endothelium-mediated changes in vascular tone mirror the downstream hyperaemic response (Schnabel et al. 2011).

IMT was assessed by measuring the common carotid artery using Duplex ultrasound, realised with the iE33 system (Philips Medical Systems, Best, The Netherlands) (Sinning et al. 2011). The measurements of the right and left side (in mm) were averaged. IMT was considered as a continuous variable.

The raw data of these subclinical parameters were evaluated by GHS physicians. Within the time frame of the project, evaluations of arterial stiffness were completed for the first 10,000 subjects; vascular function and IMT evaluations were completed for the first 5000 subjects. This resulted in a total number of valid arterial stiffness measurements for 7938 subjects, and likewise, there were *n* = 2387 valid vascular function tests and *n* = 2508 valid IMT measurements.

### Confounder and covariate selection

Scientific evidence supporting the existence of the causal pathways between relevant potential confounders for night shift work and vascular outcomes was collected. Based upon this literature overview, relevant sets of confounders for night shift work and the cardiovascular outcomes were identified using acyclic directed graphs (Textor et al. 2011).

The following covariates were included block by block in the different regression models:

- basic confounders (age and sex),
- current occupational exposures (job complexity level, supervisor/manager, overtime work, occupational noise), and
- lifestyle factors (smoking, alcohol, waist to hip ratio), SES, and dispositional factors (menopause status, family history of MI or stroke).

There is evidence to consider physical activity and vascular outcomes as confounders. Physical activity was not entered into model (c) because the recalculation to metabolic units based on the 66-item questionnaire Short QUestionnaire to ASsess Health-enhancing physical activity (SQUASH; (Campbell et al. 2016) was not finished at the time of the data analysis. Hypertension/blood pressure was not included as a confounder because it is assumed to lie on the biological pathway between shift work and the considered outcomes (Puttonen et al. 2010; Ramin et al. 2015).

*Age and sex* (a) were included in all models with age used in decades. *Current occupational exposures* (b) were coded based on the current job title and a short description of the employee’s working tasks. For this purpose, the hierarchically structured system of the German Classification of Occupations 2010 (KldB 2010) was used (Prigge et al. 2014). The fifth digit of the KldB 2010 code represents four *job complexity level:* low (helpers), medium (skilled workers; used as a reference category), complex (specialists), and very complex (experts) requirements. The dichotomous variable of being a s*upervisor*/*manager*, coded by the fourth KldB 2010-digit, reflects job position. *Overtime work* was assessed by the question ‘On average, how many hours per week are you working overtime?’, and was used continuously in hours. *Occupational noise* was inquired with a question whether the employee performs a job with high noise pollution (yes/no), followed by taking into account several noise sources (1 = refrigerator, …, 5 = pneumatic hammer) to which the workplace’s loudness level is best comparable. An affirmation of the binary request in combination with sound intensities that equal values between 3 and 5 was interpreted as being occupational noise exposed (secondary binary noise variable).

The included *lifestyle factors* (c) were smoking, alcohol, and waist to hip ratio. Smoking adjustment was based upon two concepts. The assignment *smoking* includes the categories ‘never smoker’, ‘ex-smoker’ with a quitting time of <2 years, ‘ex-smoker’ with a quitting time of more than 2 years, and ‘current smoker’. To estimate *pack*-*years*, the smoking history was enquired including its duration and the type of tobaccos (filter tips, cigarettes, cigars, tobacco, and pipe). The three categories of ‘<20’, ‘20–39’, and ‘40 or more pack-years’ were used (Jha et al. 2013; van Amelsvoort et al. 2006). For *alcohol* consumption, the categories ‘no alcohol intake’, ‘beneath tolerable limit’, ‘above tolerable limit’, and ‘abuse’ were distinguished (Burger and Mensink 2003). Waist to hip ratio was differentiated into ‘normal ratio’, ‘overweight’, and ‘obesity’, according to WHO criteria (WHO 2000).

Menopause status was queried binary (yes/no). Socio-economic status (SES) was used as an index score comprising school education, professional education, occupational position, and salary (Lampert and Kroll 2009). The scale ranges from 3 to 21 points. For the descriptive visualisation of the population, the empirically recommended cut points of <7.8 (low SES), 7.8–14 (intermediate SES), and >14 (high SES) were used (Lampert et al. 2013).

### Statistical analyses

To describe the study population and the subclinical parameters, the following analyses were performed: for continuous variables, average values, standard deviations, medians, and quartiles were used and for categorical variables, number and percentage. The population was stratified by current night shift workers, employees without night shift work, and currently unemployed subjects. Stratification by sex was applied to describe details of night shift work. Analyses for cumulative night shift were based on the total sample, whereas only actively working subjects were considered regarding current night shift work (*n* = 8065).

Linear regression models were used to estimate the effect of current and cumulative night shift work on arterial stiffness (m/s), vascular function (index points), and IMT (mm) by using generalised linear models with normal distribution and Wald statistics. The assumptions of a linear model were proved by visual control, tests on normal distribution, and correlation coefficients.

Based on the above-mentioned confounders and covariates, four different models were calculated for each outcome separately: an unadjusted, crude model (model 0); a basic model including adjustment for age and sex (model 1); a model including age and sex + occupational variables (model 2); and a model based on the adjustments of model 2 as well as of lifestyle, SES, and dispositional characteristics (model 3). The results will focus on model 2 as it contains the most relevant variables: Age and sex as well as occupational influences expressed by job complexity level, being a manager, overtime work, and noise. Model 3 will be considered as a conservative approach that covers influences of a broad spectrum, partly correcting for similar influences twice.

All variables within one confounder block were entered into the model simultaneously. The level of ‘skilled workers’ served as reference group and was contrasted to each of the three other complexity levels, respectively. Metric confounders and covariates were centred to ensure an interpretation of the intercept as an average level of the outcome at the centred values of the continuous independent variables (such as the centring of the variable age results in a value of 0 for a person aged 50). The analyses were performed by using the software R (R Core Team 2015).

For reasons of visualisation, based on the regression models, percentages of increase in arterial stiffness (m/s) were calculated for categories of night shift work exposure in the previous 10 years (exposed group) compared to the unexposed reference group by using the estimated beta coefficients and 95 % CI. This was done by the following formula: (predicted value of the exposed group − the predicted value of the unexposed group) divided by the unexposed group times 100. The predicted values of the unexposed group for all outcomes (the intercepts) are provided in the ‘Appendix’. Values of the exposed group are given in Table 1. To estimate the dose–response relationship of cumulative night shift work and arterial stiffness, a post hoc linear trend test based on the results of the regression analyses was applied. This was performed by using the package ‘generalised least squares for trend estimation of summarised dose–response data (glst)’ within Stata^®^ 13 (Orsini et al. 2009).

**Table 1:** Characteristics of current night shift workers, individuals without current night shift, currently unemployed subjects (e.g. unemployed, on parental leave, or in training), and the whole sample of ever employed subjects within the working population of the Gutenberg Health Study (*n* = 10,475)

	Current night shift (*n* = 1071)	No current night shift (*n* = 6994)	Currently unemployed (*n* = 2410)	Ever employed (*n* = 10,475)
Sex				
Males	74.2 %	51.8 %	35.1 %	50.2 %
Age (years)				
35–44	37.7 %	33.3 %	16.5 %	29.9 %
45–54	42.6 %	41.1 %	18.1 %	36.0 %
55–64	19.7 %	25.6 %	65.4 %	34.1 %
Waist hip ratio^a^				
Normal ratio	17.3 %	19.6 %	12.7 %	17.8 %
Increased risk	46.4 %	41.4 %	29.8 %	39.2 %
High risk	36.4 %	39.0 %	57.5 %	43.0 %
Smoking status				
Never	38.1 %	44.2 %	43.8 %	43.4 %
Ex-smoker (0–2 years ago)	3.6 %	2.6 %	2.0 %	2.6 %
Ex-smoker (>2 years ago)	29.0 %	30.3 %	33.8 %	31.0 %
Current smoker	29.2 %	22.9 %	20.4 %	23.0 %
Cumulative smoking				
<20 pack-years	46.6 %	44.6 %	44.6 %	44.8 %
20–39 pack-years	9.8 %	6.6 %	6.3 %	6.9 %
>40 pack-years	3.3 %	2.4 %	3.5 %	2.8 %
Alcohol intake				
No intake	45.1 %	42.1 %	49.2 %	44.0 %
Intake beneath tolerable limit^b^	31.6 %	31.2 %	25.1 %	29.9 %
Intake above tolerable limit	20.2 %	24.2 %	22.3 %	23.4 %
Abuse of alcohol	3.1 %	2.4 %	3.5 %	2.7 %
Socio-economic status				
Low	6.7 %	5.0 %	15.6 %	7.6 %
Intermediate	53.6 %	48.4 %	62.0 %	52.1 %
High	39.7 %	46.6 %	22.4 %	40.3 %
Subclinical parameters				
Arterial stiffness (m/s) (median [25/75 quartiles])	8.2 (6.3/10.8)	7.5 (6.0/10.1)	8.3 (6.3/10.8)	7.7 (6.1/10.4)
Vascular function^c^ (mean value [SD])	0.57 (0.39)	0.64 (0.40)	0.64 (0.40)	0.63 (0.40)
Intima media thickness (mm) (median [25/75 quartiles])	0.59 (0.53/0.67)	0.58 (0.53/0.66)	0.64 (0.56/0.72)	0.60 (0.54/0.68)

^a^Limits for men: normal ratio <0.9; increased risk ≥0.9 and ≤1; high risk >1; limits for women: normal ratio <0.8; increased risk ≥0.8 and ≤0.85; high risk >0.85

^b^Limits for men: beneath tolerable limit ≤20 g/day; above tolerable limit >20–60 g/day; abuse >60 g/day; limits for women: beneath tolerable limit ≤10 g/day; above tolerable limit >10–40 g/day; abuse >40 g/day

^c^Reactive hyperaemia index (RHI)

## Results

### Description of the study population

Night shift work was performed by 1071 out of 8065 currently employed individuals with 7.6 % of the women and 18 % of the men operating nights. Table 1 characterises the sample population under investigation. It consists of current employed individuals (night shift workers and individuals without current night shift), currently unemployed subjects (e.g. unemployed, on parental leave, or in training), and the whole sample of ever employed subjects within the GHS working population.

Current night shift workers differed from those currently not working at night with regard to several characteristics: the percentage of males (74.2 %) was high, and night workers formed the group with the largest proportion of younger employees. The proportion of current night workers that were overweight slightly exceeded the fraction of those in day work as well as currently unemployed individuals. The highest proportion of current smokers (29 %) and heavy smokers (13 % had more than 20 pack-years) was also observed among subjects currently working at night. There are also differences between the groups according to SES (Table 1).

On average, within the individuals that work at night, both men and women carried out seven night shifts in the last month preceding the investigation. Typical sex differences surfaced particularly when cumulative night work exposure was considered. Within the last decade, men operated on average 116 nights, whereas women carried out an average of 53 nights shifts. Approximately the same sex divergence is true over the entire professional life with an average of 356 nights in shift work for men versus 151 night shifts for women. The sum of night shifts carried out over the active life equalled approximately the threefold number of the last 10 years.

The unadjusted distribution of subclinical parameters (Table 1) showed an elevated arterial stiffness for subjects operating at night compared to individuals without current night shift work. The RHI, describing vascular function, was lower in subjects currently working at night, thus pointing to an advanced atherosclerotic process in night shift workers. Similarly, numerically night shift workers had a slightly higher IMT compared to individuals not working between 11 p.m. and 5 a.m. Individuals without current employment showed numerically the most unfavourable values concerning IMT and arterial stiffness, whereas this was not the case for vascular function (Table 1).

### Current night shift

Results of the linear regression analyses confirmed an effect of night shift in the current job on arterial stiffness with a significant increase of 0.02–0.06 m/s per currently operated night (depending on the adjustment, Table 2). When applied to individuals with seven night shifts per months, this equals an increase of 0.21 m/s (95 % CI 0.07–0.35 m/s) after adjustment for age and sex, job complexity level, being a manager, overtime work, and noise (model 2). Arterial stiffness increased by approximately 3 %.

**Table 2:** Association of current night shift with subclinical atherosclerosis. Estimated beta coefficients for arterial stiffness, vascular function, and intima media thickness (95 % confidence interval) within the currently employed working population of the Gutenberg Health Study (*n* = 8065 subjects aged 35–64 years)

Exposure	Marker of subclinical atherosclerosis (number of available measurements)					
	Current night shift work					
	Arterial stiffness (*n* = 5982) effect estimate: beta coefficient (m/s)		Vascular function [reactive hyperaemia index (RHI)] (*n* = 1787) effect estimate: beta coefficient (index points)		Intima media thickness (*n* = 1868) effect estimate: beta coefficient (mm)	
	Night shift yes versus no	Number of night shifts per month	Night shift yes versus no	Number of night shifts per month	Night shift yes versus no	Number of night shifts per month
Model 0 unadjusted	**0**.**45** (**0**.**25**/**0**.**66**)	**0**.**06** (**0**.**04**/**0**.**09**)	−**0**.**074** (−**0**.**127**/−**0**.**022**)	−**0**.**007** (−**0**.**013**/−**0**.**001**)	−0.002 (−0.016/0.012)	0.000 (−0.002/0.001)
Model 1^a^	0.17 (−0.01/0.36)	**0**.**04** (**0**.**02**/**0**.**06**)	0.030 (−0.081/0.020)	−0.004 (−0.009/0.002)	0.004 (−0.009/0.016)	0.000 (−0.001/0.001)
Model 2^b^	0.08 (−0.10/0.26)	**0**.**03** (**0**.**01**/**0**.**05**)	−0.025 (−0.076/0.027)	−0.003 (−0.009/0.003)	0.002 (−0.022/0.014)	0.000 (−0.002/0.001)
Model 3^c^	0.03 (−0.15/0.21)	**0**.**02** (**0**/**0**.**04**)	−0.018 (−0.068/0.033)	−0.003 (−0.009/0.003)	0.000 (−0.012/0.013)	0.000 (−0.002/0.001)

^a^Model 1: adjusted for age and sex

^b^Model 2: adjustments of model 1 + occupational variables (+job complexity level, being a manager, overtime work, noise)

^c^Model 3: adjustments of model 2 + socio-economic status, smoking status, pack-years, alcohol intake, waist to hip ratio, menopause status, and family history of myocardial infarction or stroke

Results in bold font indicate confidence intervals that do not contain the null hypothesis value

An effect of working at night (yes vs. no) as well as of the number of nights spent working on the vascular function was not statistically significant after adjustment (Table 2; *p* values for model 2 *p* = 0.35 for night shift work yes versus no and *p* = 0.27 for the number of nights). As Table 2 shows, regression analyses did also not support relevant associations of current shift work and IMT.

### Cumulative night shift

Results of the regression analyses showed an effect of cumulative night shift work on arterial stiffness. For the high exposure group with more than 660 night shifts, there were significant increases of 0.23 and 0.33 m/s of arterial stiffness associated with cumulative exposure within the last 10 years (Table 3, model 2) and the entire professional life (Table 4, model 2). Compared to the average arterial stiffness of individuals without night shift work (the reference group), this reflects increases of 3 and 4 %, respectively.

**Table 3:** Association of cumulative number of night shifts within the last 10 years (database are 10,475 ever employed individuals)

Exposure	Marker of subclinical atherosclerosis (number of available measurements)								
	Cumulative number of night shifts within the last 10 years								
	Arterial stiffness (*n* = 7938) effect estimate: beta coefficient (m/s)			Vascular function [reactive hyperaemia index (RHI)] (*n* = 2387) effect estimate: beta coefficient (index points)			Intima media thickness (*n* = 2508) effect estimate: beta coefficient (mm)		
Categories (nights)	1–220 versus 0	221–660 versus 0	>660 versus 0	1–220 versus 0	221–660 versus 0	>660 versus 0	1–220 versus 0	221–660 versus 0	>660 versus 0
Model 0 unadjusted	0.25 (−0.04/0.55)	**0**.**41** (**0**.**12**/**0**.**71**)	**0**.**88** (**0**.**57**/**1**.**19**)	−0.053 (−0.124/0.018)	−**0**.**120** (−**0**.**194**/−**0**.**045**)	−**0**.**096** (−**0**.**173**/−**0**.**020**)	−0.008 (−0.027/0.012)	−0.013 (−0.034/0.008)	−0.004 (−0.025/0.017)
Model 1^a^	0.19 (−0.07/0.45)	0.26 (0.00/0.51)	**0**.**48** (**0**.**21**/**0**.**75**)	−0.019 (−0.087/0.049)	−**0**.**077** (−**0**.**148**/−**0**.**006**)	−0.038 (−0.111/0.035)	0.005 (−0.012/0.021)	−0.007 (−0.024/0.011)	0.007 (−0.011/0.024)
Model 2^b^	0.18 (−0.08/0.44)	0.22 (−0.04/0.48)	**0**.**33** (**0**.**06**/**0**.**61**)	−0.019 (−0.087/0.049)	−0.070 (−0.142/0.001)	−0.035 (−0.110/0.039)	0.006 (−0.011/0.022)	−0.008 (−0.026/0.009)	−0.002 (−0.016/0.020)
Model 3^c^	0.11 (−0.15/0.36)	0.21 (−0.04/0.47)	0.23 (−0.04/0.5)	−0.004 (−0.071/0.062)	−**0**.**072** (−**0**.**142**/−**0**.**001**)	−0.028 (−0.101/0.046)	0.003 (−0.013/0.019)	−0.008 (−0.025/−0.010)	−0.002 (−0.020/0.016)

Estimated beta coefficients for arterial stiffness, vascular function, and intima media thickness (95 % confidence interval) within the working population of the Gutenberg Health Study (*n* = 10,475 subjects aged 35–64)

^a^Model 1: adjusted for age and sex

^b^Model 2: adjustments of model 1 + occupational variables (+job complexity level, being a manager, overtime work, noise)

^c^Model 3: adjustments of model 2 + socio-economic status, smoking status, pack-years, alcohol intake, waist to hip ratio, menopause status, and family history of myocardial infarction or stroke

Results in bold font indicate confidence intervals that do not contain the null hypothesis value

**Table 4:** Cumulative number of night shifts throughout the entire professional life (database are 10,475 ever employed individuals)

Exposure	Marker of subclinical atherosclerosis (number of available measurements)								
	Cumulative number of night shifts throughout the entire professional life								
	Arterial stiffness (*n* = 7938) effect estimate: beta coefficient (m/s)			Vascular function [reactive hyperaemia index (RHI)] (*n* = 2387) effect estimate: beta coefficient (index points)			Intima media thickness (*n* = 2508) effect estimate: beta coefficient (mm)		
Categories (nights)	1–220 versus 0	221–660 versus 0	>660 versus 0	1–220 versus 0	221–660 versus 0	>660 versus 0	1–220 versus 0	221–660 versus 0	>660 versus 0
Model 0 unadjusted	**0**.**45** (**0**.**23**/**0**.**68**)	**0**.**46** (**0**.**23**/**0**.**7**)	**1**.**00** (**0**.**80**/**1**.**2**)	−0.043 (−0.096/0.010)	−**0**.**078** (−**0**.**138**/−**0**.**018**)	−**0**.**140** (−**0**.**191**/−**0**.**090**)	−0.010 (−.025/0.005)	0.002 (−0.014/0.019)	**0**.**017** (**0**.**003**/**0**.**031**)
Model 1^a^	0.12 (−0.08/0.32)	**0**.**22** (**0**.**01**/**0**.**43**)	**0**.**33** (**0**.**15**/**0**.**51**)	0.042 (−0.010/0.094)	−0.035 (−0.092/0.023)	−**0**.**058** (−**0**.**108**/−**0**.**009**)	0.000 (−0.013/0.012)	0.005 (−0.009/0.019)	0.009 (−0.003/0.021)
Model 2^b^	0.14 (−0.06/0.35)	0.20 (−0.01/0.41)	**0**.**23** (**0**.**05**/**0**.**42**)	0.040 (−0.013/0.092)	−0.033 (−0.091/0.024)	−**0**.**054** (−**0**.**104**/−**0**.**003**)	0.001 (−0.011/0.014)	0.005 (−0.009/0.019)	0.006 (−0.006/0.018)
Model 3^c^	0.10 (−0.09/0.3)	0.17 (−0.04/0.38)	0.15 (−0.03/0.33)	0.044 (−0.008/0.095)	−0.038 (−0.095/0.019)	−0.042 (−0.091/0.008)	0.000 (−0.012/0.013)	0.005 (−0.009/0.018)	0.002 (−0.010/0.015)

Estimated beta coefficients for arterial stiffness and vascular function, and intima media thickness (95 % confidence interval) in subjects aged 35–64

^a^Model 1: adjusted for age and sex

^b^Model 2: adjustments of model 1 + occupational variables (+job complexity level, being a manager, overtime work, noise)

^c^Model 3: adjustments of model 2 + socio-economic status, smoking status, pack-years, alcohol intake, waist to hip ratio, menopause status, and family history of myocardial infarction or stroke

Results in bold font indicate confidence intervals that do not contain the null hypothesis value

**Fig. 1:**
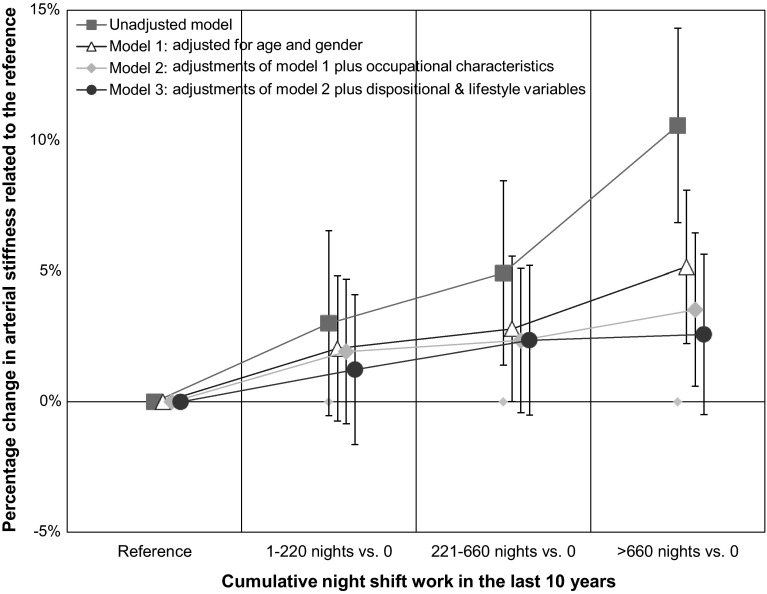
Association between cumulative night shift work during the last 10 years and arterial stiffness (in four statistical models). Percentage change in arterial stiffness and 95 % confidence intervals (CI) based on the ratio of subjects exposed to night shift and the unexposed reference group (basis of the comparisons: beta estimates (m/s) and 95 % CI)

Figure 1 depicts the relationship between cumulative night shift work within the last decade and arterial stiffness. Higher cumulative night shift exposures were associated with a more pronounced arterial stiffness. A dose–response relationship was indicated by a statistically confirmed increase only for the unadjusted model and shows a linear increase in the pulse wave velocity of 0.26 m/s (0.39–4.89 m/s) per night shift work category.

For vascular function, significant decreases in the RHI were confirmed for exposures of 221–660 nights spent working within the last 10 years as well as for the highest exposure of night work within the active life (>660 nights). This corresponds to a decrease in the RHI by approximately 12 % per night shift work category for night shift workers compared with the estimated value for unexposed subjects.

Neither cumulative exposure to night shift work within the last 10 years nor throughout the entire work life was significantly associated with the IMT.

### Sensitivity analysis

Neither the exclusion of subjects with a pre-existing CVD nor the additional exclusion of persons previously diagnosed with cancer had an effect on the results (data not shown).

## Discussion

In summary, the presented analyses of the baseline data from a large population-based study show an impact of the cumulative number of night shifts throughout the entire professional life on the process of atherosclerosis as indicated by increased arterial stiffness and decreased vascular function. In addition, arterial stiffness was associated with cumulative number of night shifts within the last 10 years and with the number of current night shifts. However, no association was found between current and cumulative night shift exposure and an increased IMT.

Several possible causal mechanisms are described explaining the link between shift work, CVDs, and the preceding process of atherosclerosis: the hypotheses include circadian disruption, sleep deprivation, and social stress related to shift work, lifestyle changes (smoking, lack of physical activity), and low SES. Each of the above-mentioned mechanisms may operate differently (e.g. inflammatory processes, circadian disruption resulting in different functions of endothelial cells, and vascular smooth muscle cells) with respect to the investigated subclinical parameters arterial stiffness, vascular function, and IMT investigated in the GHS. This may contribute to the inconsistent results.

Within the three outcomes considered, arterial stiffness displayed both the strongest effects and a dose–response relationship with cumulative night shift work. Arterial stiffness is a functional marker with a very good reproducibility (Meyer et al. 2016). The underlying mechanisms for the null result regarding the association between night shift work and the structural parameter IMT are difficult to explain. In contrast to the other two subclinical markers considered, IMT was categorised and used as a binary variable. However, models with IMT as a continuous variable gave similar results (data not shown). One explanation is the large standard deviation of the measurement compared to arterial stiffness.

Furthermore, three points related to the exposure need to be taken into account for the interpretation of the results. First, in GHS, like in most population-based cohort studies on shift work, self-reported information on the occupational exposure without validation by objective company data is used. Second, the exposure under investigation was night shift requested as ‘working between 11 p.m. and 5 a.m.’. This description of the exposure is distinct. On the other hand, it may give rise to misclassification, since employees with evening shift until 12 p.m. can be wrongly graded as night shift workers. Third, the unexposed reference group in the analyses on cumulative night shift exposure included a considerable percentage of persons with periods of unemployment. Currently, unemployed subjects within the study population were characterised by accumulated cardiovascular risk factors, such as higher age, a lower SES, and a higher number of subjects with obesity. This means a higher likelihood for the presence of atherosclerosis. Thus, the presented results obtained by regression analyses investigating the effect of cumulative night shift exposure should be rated as conservative.

Discrepancies between the results found with different statistical models may partly be explained by the inclusion of cardiovascular risk factors such as body proportion and smoking as covariates, which may rather be intermediate variables between shift work and CVDs, and thus attenuate the effect. Many studies support an association of shift work with changes in lifestyle factors such as weight gain (Amani and Gill 2013). Furthermore, night workers already smoke more before they enter into shift work (Nabe-Nielsen et al. 2008). As smoking status and pack-years were not included in our main model, residual confounding cannot be ruled out. However, the association between current number of night shifts per month and arterial stiffness remained significant in the model that additionally controlled for lifestyle factors including smoking. In general, since covariates were not separately included into the models, but in blocks, a contribution of single mediating factors or confounders is difficult to conclude.

### Comparison with results described in the literature

Measurements of the investigated subclinical markers have sporadically been included in investigations considering occupational exposures, but only few investigations consider the relation between shift work and these subclinical parameters.

#### Arterial stiffness

A pilot study investigating pulse wave velocity in relation to shift work in male steelworkers found no significant differences between day workers and shift workers with respect to arterial stiffness. However, there was a positive correlation between the individual shift workload (a measure including social jetlag, speed of shift work rotation, and years of shift work rotation) (Kantermann et al. 2013) and arterial stiffness. This indication of a dose–response relationship resembles the results of the present study. Also, an investigation of professional bus drivers exploring brachial ankle pulse wave velocity in shift drivers versus non-shift drivers found a higher pulse wave velocity in long-term shift drivers compared to regular and short-term shift drivers (Chen et al. 2010).

#### Vascular function

Vascular function in relation to shift work was considered in several small-scale studies investigating different occupational groups, particularly medical staff, by applying different techniques. Despite the different techniques, all results support an association of shift work with impaired vascular function. The presented analyses from a large population-based study are generally in line with these results. The inconsistencies within the present results may be due to a smaller sample size, leading to a loss of power. Estimates may fail to reach statistical significance as compared with the presented analyses of arterial stiffness. Yet, the sample size for analyses of vascular function of the current GHS study exceeds those included in previous studies. A study investigating healthy medical residents, without any cardiovascular risk factor, found slightly impaired flow-mediated dilation (FMD) after a working night compared to a restful night (Tarzia et al. 2012). Amir et al. (2004) reported an abnormal brachial artery endothelial function in residents and house staff after a 24-h shift, including night duty and described dose–response relationships: the largest decrease in FMD was observed in physicians with a longer history of night shift duty and in those reporting fewer sleeping hours during the shift. Also, results of the GHS show the most distinct change in vascular function in the group of employees who had the highest shift work exposure considering their total working life. Suessenbacher et al. (2011) investigated 48 male shift workers and 47 male non-shift workers of a glass manufactory and indicated a reduced vascular function. Dispatchers in shift work exhibited reduced vascular functioning (Wong et al. 2012).

#### Intima media thickness

In contrast to arterial stiffness and vascular function, the presented analyses did not show a relation between shift work exposure and an increase in the IMT after adjustment.

However, results from the literature are ambiguous. A cross-sectional study by Haupt et al. (2008) describes a higher atherosclerotic risk in shift workers aged at least 45 years showing an increased IMT within a logistic (instead of a linear) model. Results of the Cardiovascular Risk in Young Finns study (Puttonen et al. 2009) also suggest that shift work is associated with an increased IMT observable in men already before age 40, but not in women. The prospective Kuopio Ischaemic Heart Disease Risk Factor Study (Wang et al. 2015) investigated the relationship between different work schedules and the 11-year progression of carotid atherosclerosis indicating that weekend shifts, more than standard daytime work, appear to accelerate carotid atherosclerosis progression among middle-aged Finnish men, especially those with pre-existing CVD. However, no substantial difference in IMT increase was found when comparing employees who did evening/night or rotating shifts with standard daytime workers.

### Limitations and strengths

The following limitations of the presented cross-sectional analysis of the GHS baseline need to be mentioned. Information about features of shift work was lacking (e.g. shift type, direction of rotation). Thus, the inquiry implies a risk for misclassification as already discussed. All information about shift work is based on retrospective self-assessments. The number of valid measurements of vascular function and IMT was limited for this baseline analysis. Therefore, the statistical power to detect effects of different levels of exposure to night shift work on these subclinical markers was reduced. It was not possible to take into account physical activity. The longitudinal analyses will consider physical activity as a potential confounder.

Several characteristics of this study are notably. Compared to the results from the literature discussed above, the number of participants investigated in the GHS is large, including different age groups of the working population as well as a variety of occupations. Information about cumulative night shift exposure based on a comprehensive occupational history was included. Additionally, three subclinical parameters have been investigated and measured with highly standardised procedures and quality control. To our knowledge, this is the first population-based study on shift work and arterial stiffness and endothelial function, respectively.

### Implications for further research

The presented results from the baseline of a large prospective cohort study (GHS) investigating several risk factors with respect to cardiovascular health indicate an effect of cumulative night shift work on the atherosclerotic process looking retrospectively on the number of night shifts during professional life. Further longitudinal analyses projected within the design of the GHS are necessary to confirm these results by taking into account a priori defined levels of clinically important changes in the investigated parameters during follow-up. The results also provide evidence for the potential usefulness of these earlier subclinical markers as outcomes in epidemiological studies on occupational cardiovascular health. Possible associations are usually examined considering incident myocardial infarction—an outcome which takes many years to develop. Future studies on cardiovascular risks of shift work could be improved with a more differentiated assessment of working time (e.g. shift intensity, length of working hours) and subclinical atherosclerosis as suggested by Härmä et al. (2015). In particular, further research is needed on the effectiveness of interventions in work organisation, e.g. changing shift rosters could be monitored by the parameters used in the present study.

Hypertension may be on the biological pathway from shift work to atherosclerosis (Puttonen et al. 2010) and was therefore not considered as a confounder. When we excluded all subjects with a self-reported diagnosis of CVD in a sensitivity analysis, the results were similar (data not shown). Future studies might wish to study the effect of shift work on atherosclerosis independent of hypertension.

### Implications for occupational health practitioners

This study indicates an increased risk of atherosclerosis in long-term night shift workers, making them a potential target group for health promotion and surveillance, including preventive medical examinations to detect early signs of CVD. It seems reasonable to integrate arterial stiffness or the measurement of vascular function in these medical examinations to monitor the atherosclerotic process in order to better assess the demanding effect of night shift work.

## Appendix

See Tables 5, 6 and 7.

**Table 5:** Intercept values of unexposed participants to complement Table 2: arterial stiffness (m/s), vascular function (index points), and intima media thickness (mm) with 95 % confidence interval (CI) of currently employed subjects without current shift work of the Gutenberg Health Study (*n* = 8065 subjects aged 35–64 years)

Exposure	Marker of subclinical atherosclerosis (number of available measurements)					
	Not exposed: no current night shift work					
	Arterial stiffness (*n* = 5982) intercept (m/s)		Vascular function [reactive hyperaemia index (RHI)] (*n* = 1787) intercept (index points)		Intima media thickness (*n* = 1868) intercept (mm)	
	Night shift yes versus no	Number of night shifts per month	Night shift yes versus no	Number of night shifts per month	Night shift yes versus no	Number of night shifts per month
Model 0 unadjusted	8.22 (8.14/8.29)	8.28 (8.21/8.35)	0.638 (0.619/0.658)	0.628 (0.609/0.646)	0.602 (0.597/0.608)	0.602 (0.597/0.607)
Model 1^a^	9.08 (8.99/9.17)	9.11 (9.02/9.19)	0.522 (0.497/0.547)	0.517 (0.494/0.541)	0.610 (0.604/0.616)	0.611 (0.605/0.617)
Model 2^b^	9.16 (9.03/9.29)	9.16 (9.04/9.29)	0.511 (0.474/0.548)	0.508 (0.472/0.544)	0.619 (0.611/0.621)	0.620 (0.611/0.628)
Model 3^c^	8.66 (8.49/8.83)	8.66 (8.50/8.83)	0.592 (0.543/0.640)	0.590 (0.542/0.638)	0.608 (0.697/0.620)	0.609 (0.697/0.620)

^a^Model 1: adjusted for age and sex

^b^Model 2: adjustments of model 1 + occupational variables (+job complexity level, being a manager, overtime work, noise)

^c^Model 3: adjustments of model 2 + socio-economic status, smoking status, pack-years, alcohol intake, waist to hip ratio, menopause status, and family history of myocardial infarction or stroke

**Table 6:** Intercept values of unexposed participants to complement Table 3: arterial stiffness (m/s), vascular function (index points), and intima media thickness (mm) with 95 % confidence interval (CI) of subjects without current shift work within the last 10 years across the 10,475 ever employed individuals aged 35–64 of the Gutenberg Health Study

Exposure	Marker of subclinical atherosclerosis (number of available measurements)		
	Not exposed: no night shift work within the last 10 years		
	Arterial stiffness (*n* = 7938) intercept (m/s)	Vascular function [reactive hyperaemia index (RHI)] (*n* = 2387) intercept (index points)	Intima media thickness (*n* = 2508) intercept (mm)
Model 0 unadjusted	8.31 (8.24/8.37)	0.640 (0.623/0.657)	0.616 (0.611/0.621)
Model 1^a^	9.29 (9.21/9.37)	0.514 (0.491/0.537)	0.626 (0.621/0.632)
Model 2^b^	9.35 (9.25/9.46)	0.496 (0.466/0.526)	0.632 (0.625/0.640)
Model 3^c^	8.88 (8.74/9.02)	0.580 (0.539/0.620)	0.619 (0.609/0.629)

^a^Model 1: adjusted for age and sex

^b^Model 2: adjustments of model 1 + occupational variables (+job complexity level, being a manager, overtime work, noise)

^c^Model 3: adjustments of model 2 + socio-economic status, smoking status, pack-years, alcohol intake, waist to hip ratio, menopause status, and family history of myocardial infarction or stroke

**Table 7:** Intercept values of unexposed participants to complement Table 4: arterial stiffness (m/s), vascular function (index points), and intima media thickness with (95 % confidence interval) of subjects without current shift work throughout the entire professional life across the 10,475 ever employed individuals aged 35–64 of the Gutenberg Health Study

Exposure	Marker of subclinical atherosclerosis (number of available measurements)		
	Not exposed: no night shift work throughout the entire professional life		
	Arterial stiffness (*n* = 7938) intercept (m/s)	Vascular function [reactive hyperaemia index (RHI)] (*n* = 2387) intercept (index points)	Intima media thickness (*n* = 2508) intercept (mm)
Model 0 unadjusted	8.20 (8.13/8.27)	0.654 (0.635/0.672)	0.614 (0.608/0.619)
Model 1^a^	9.26 (9.17/9.35)	0.511 (0.485/0.537)	0.625 (0.618/0.631)
Model 2^b^	9.33 (9.22/9.44)	0.496 (0.464/0.528)	0.631 (0.623/0.639)
Model 3^c^	8.86 (8.72/9.01)	0.578 (0.537/0.620)	0.618 (0.607/0.628)

^a^Model 1: adjusted for age and sex

^b^Model 2: adjustments of model 1 + occupational variables (+job complexity level, being a manager, overtime work, noise)

^c^Model 3: adjustments of model 2 + socio-economic status, smoking status, pack-years, alcohol intake, waist to hip ratio, menopause status, and family history of myocardial infarction or stroke
